# Perspective: Emerging Challenges in the Treatment of Influenza and Parainfluenza in Transplant Patients

**DOI:** 10.1155/2011/910930

**Published:** 2011-07-07

**Authors:** Ronald B. Moss, Roy T. Steigbigel, Rebecca L. Sanders, Fang Fang

**Affiliations:** ^1^NexBio Inc., 10665 Sorrento Valley Road, San Diego, CA 92121, USA; ^2^Stony Brook University School of Medicine, Stony Brook, New York, NY 11794, USA

## Abstract

Influenza, respiratory synctial virus, and parainfluenza are common respiratory infections in immunocompromised transplant recipients, causing significant morbidity and mortality in this patient population. This paper focuses on influenza and parainfluenza virus infections in transplant patients with emphasis on the pandemic 2009 H1N1 influenza infection. Current antiviral treatment recommendations for influenza and parainfluenza in immunocompromised patients as well as novel investigational therapeutic approaches currently being tested in the clinic are discussed. In addition to the morbidity and mortality caused by these viruses, the development of multidrug resistance leading to transmission of resistant viruses is of great public health concern. The development of effective new therapies for influenza and parainfluenza in these high-risk patients is needed with randomized placebo-controlled studies to assess their clinical utility.

## 1. Introduction

Preventing and treating infectious diseases in immunocompromised patients presents special challenges, as current treatments and vaccines may have limited efficacy in this population. Immune suppression is an essential component of successful solid organ and hematopoietic cell transplantation (SOT and HCT, resp.). SOT and HCT transplant recipients require immune suppressants during the first 100 days posttransplant period (e.g., FK506, cyclosporine, prednisone) in order to prevent graft rejection and minimize graft-versus-host disease (GVHD). SOT patients require immunosuppression indefinitely. Recipients of HCT and SOT, undergo ablation of the immune system prior to transplantation, and immune reconstitution occurs only after successful engraftment of the transplant. Therefore, SOT and HCT patients are severely immunocompromised for a significant period and are at high-risk for various opportunistic infections that can cause significant morbidity and mortality. 

Influenza (IFV), respiratory synctial virus (RSV), and parainfluenza (PIV) are common respiratory infections in both immune competent and immunocompromised populations. This paper will focus on influenza and parainfleunza virus infections in transplant populations. The recent emergence of the novel 2009 H1N1 (2009H1N1v) influenza virus has illustrated many challenges in preventing and treating respiratory viral infections and underscores the need for appropriate combative therapies for this at risk population. Some of these challenges include the lack of population immunity to a novel IFV strain that led to pandemic outbreaks as well as the potential for the development of drug resistance that has rendered existing therapeutic modalities less effective [1, 2]. Our search strategy for this paper included the National Library of Medicine (PubMed) and manufacturers trial data bases available on the internet. 

 While sharing similarities, infections caused by IFV and PIV differ in a number of important ways, noted in Table 1. IFV and PIV are genetically distinct, single-stranded RNA viruses of the Orthomyxoviridae and Paramyxoviridae families, respectively. Both are tropic for the human respiratory tract and utilize sialic acid as their receptors [3–5]. While influenza is usually a seasonal disease, parainfluenza occurs throughout the year [6]. Vaccination is an important and effective approach to preventing influenza in the immunocompetent host. However, efficacy of vaccines is generally reduced in HCT patients, particularly during the first 100 days post transplant, due to iatrogenic immune suppression [7]. A recent report noted that only 51% of HCT patients receiving influenza vaccine had adequately protective antibody titers of 1:40 or higher after vaccination [8]. In addition, a review of over 40 studies of SOT noted a reduction in efficacy of influenza vaccination in this population compared to healthy controls [9]. Antivirals are available to treat influenza infections but efficacy has not been definitively demonstrated in the transplant recipients. Even with current antiviral therapy, IFV infections in transplant recipients are characterized by prolonged viral shedding leading to the risk of developing drug resistant strains. PIV is even more problematic as there are neither antiviral drugs nor vaccines available to treat or prevent this infection. 

## 2. Influenza Virus Infection in the Immunocompromised Host

Influenza is typically caused by infection with either influenza A virus (IFV A) or influenza B virus (IFV B) each composed of 11 genes encoded by 8 negative-stranded RNA segments enclosed in a lipid envelope derived from the host cell. The envelope displays three key viral proteins: hemagglutinin (HA) attaches the virus to host cell receptors and mediates fusion of viral and cellular membranes; neuraminidase (NA) facilitates release of new viruses from the host cell, and M2 proteins serve as ion channels. Only influenza A (IFVA) viruses are further classified by subtype on the basis of the two main surface glycoproteins HA and NA. There are 16 known HA subtypes and 9 known NA subtypes of IFV A. Subtypes of influenza A that are currently circulating worldwide include 2009H1N1v, seasonal H1N1 and H3N2v. Approved antiviral drugs target two of the three above described viral proteins, M2 and NA. They include the M2 inhibitors adamantanes (amantadine and rimantadine), and the NA inhibitors (NAI) oseltamivir (Tamiflu), peramivir, and zanamivir (Relenza).

The impact of seasonal influenza on immunocompromised individuals has been clinically characterized primarily through case studies. Complications from influenza in this population include high rates of mortality, a need for mechanical ventilation, progression to lower airway disease, high rates of secondary bacterial infection, and persistent viral shedding [10, 11]. Morbidity and mortality from influenza is higher in immunocompromised individuals compared to immunocompetent patients [10]. Lymphopenia appears to be an important risk factor for developing lower airway disease such as pneumonia and mortality after progression to pneumonia can be as high as 30% [10]. 

 There are multiple causes for the increased susceptibility to influenza infection in immunocompromised patients. In HCT, the intensity of myeloablation and consequent lymphopenia, increase susceptibility to respiratory infections including influenza [12]. IFV in the immunocompetent individual usually results in viral shedding that is self-limited, lasting five to six days. In contrast, viral shedding prolonged for over 2 weeks is common in immunocompromised hosts and has reportedly lasted as long as six months [13, 14]. Prolonged viral replication and shedding of IFV has been associated with selection of resistant virus. For example, in a small series of HCT subjects, the incidence of NAI resistance was 67% [15]. Additional studies suggest that the incidence of resistance is higher in immunocompromised patients than in immunocompetent adults or children [16, 17]. 

## 3. 2009H1N1v Infection

Emergence of the pandemic 2009H1N1v unveiled many of the challenges in preventing and treating respiratory viral infections. The 2009H1N1v originated from genetic reassortment between IFVs from humans, birds, and pigs. Both of the FDA-approved adamantanes lost inhibitory activity towards the M2 channel of 2009H1N1v, due to mutation in amino acid Ser31 [18, 19]. The M gene encoded by this new pandemic influenza virus is reportedly similar to the M gene in the Eurasian Swine virus, which confers resistance to both amantadine and rimantidine. Fortunately, the majority of 2009H1N1v isolates tested to date do remain sensitive to the NAIs. However, influenza drug resistance to 2009H1N1v has been described in the immunocompetent host. As of August 2010, the World Health Organization had documented 304 cases associated with the H274Y mutations (histidine to tyrosine at codon 274 in N2 nomenclature or H275Y in N1 nomenclature) in H1N1v with reduced sensitivity to oseltamivir [20, 21]. The concern for the potential for increased resistance is also exemplified by a recent report of several genetic changes in 2009H1N1v isolates from the Southern Hemisphere that have been associated with vaccine breakthroughs and a number of fatalities in both Singapore and Australia [22]. Thus there is concern that drug resistance could become prevalent, as occurred with previous seasonal IFV strains in 2009, when mutations associated with resistance to oseltamivir were found in almost all isolates [23]. 

Many case reports available have documented significant morbidity and mortality in transplant patients infected in 2009H1N1v, and underscores the need to monitor the emergence of new pandemic strains as well as the development of viral resistance in these particular patient populations.

A number of cases of severe 2009H1N1v infection resulting in significant morbidity and mortality have been described in HCT and SOT patients and in some they were associated with drug resistance. A recent report of 237 cases of 2009H1N1v in SOT and found that thirty-two percent had pneumonia [24]. Sixteen percent were admitted to intensive care and four percent died. Most patients received oseltamivir and there was some clinical resolution from early treatment with antivirals. 

Another study examined the outcome of 27 H1N1v patients postHCT [25]. Influenza-related 30-day overall mortality was 22% while patients with lower respiratory tract infection (LRTI) had a 43% mortality rate. Chronic steroid use (≥20 mg/day of prednisone equivalent) at the time of presentation was a risk factor for LRTI and death. 

Possible risk factors and poorer outcomes were examined in 13 patients with 2009H1N1v infection post HCT [26]. Five of 13 patients had 2009H1N1 influenza-induced LRTI and only 1 survived. The authors noted that lower respiratory tract disease and poorer outcomes occurred in patients receiving intense immunosuppressive therapy who were neutropenic and had GVHD. 

Antón et al. described the development of drug resistance and the associated viral kinetics after infection with 2009H1N1v in an immunocompromised patient [27]. Resistance to oseltamivir was observed after 10 days of treatment. During subsequent treatment with zanamivir, viral loads remained elevated for 5 days but then declined over an additional 7 days. 

The Centers for Disease Control (CDC) described infections in two immunocompromised individuals early in the 2009H1N1v pandemic [28]. The first patient developed influenza-like symptoms approximately 30 days after HCT, and was treated with oseltamivir for >6 weeks, with evidence of persistence infection by PCR assay. Sequence analysis revealed variants containing H274Y mutation within two weeks after beginning oseltamivir treatment began. A second patient developed respiratory symptoms after two cycles of chemotherapy and was initially treated with oseltamivir and rimantadine. H274Y was detected and oseltamivir discontinued. Treatment with inhaled zanamivir was attempted but poorly tolerated. The patient was subsequently treated with intravenous zanamivir and ribavirin in combination. She remained symptomatic at the time of the report with no additional followup on the patient's clinical status. 

Memmoli et al. described two HCT patients who rapidly developed resistance to oseltamivir and peramivir after infection with 2009H1N1v [29]. One developed respiratory symptoms and was treated for 30 days with oseltamivir. The H274Y mutation was detected by day 9 posttreatment. The other developed mild upper respiratory symptoms and was treated with oseltamavir. After 24 days of continuous oseltamivir therapy the patient developed respiratory distress and bronchoscopy revealed the presence of IFV. The patient then received 10 days of inhaled zanamivir with symptomatic improvement. 

Selection of multidrug resistance to all available NAI's (oseltamivir, zanamivir, and peramivir) was described in a pediatric patient who developed influenza-like symptoms just prior to HCT [30]. Influenza was confirmed by PCR and the patient was treated with oseltamivir. Twelve days later the H274Y mutation was detected and the patient received zanamivir intravenously. The viral load decreased and the patient was discharged but returned approximately 3 weeks later with upper respiratory symptoms. Intravenous zanamivir was reinitiated, however the virus persisted and a new mutation, I223R, was detected on day 55, exhibiting decreased sensitivity to oseltamivir, zanamivir, and peramivir. Respiratory status of this patient worsened, eventually leading to death. 

Renuad et al., described a patient who developed respiratory symptoms and fever approximately 2 years after allogeneic HCT [31]. The patient received oseltamivir, but his respiratory status declined and bronchoalveolar lavage fluid revealed a high viral load for 2009H1N1v. PCR revealed that >90% of viral RNA was wild-type, encoding histidine at position 274. On day 7 the patient began intravenous peramivir. However, on day 17, because of continued viral shedding, peramivir was discontinued and H274Y confirmed as present by PCR, illustrating the emergence of resistance to NAI's during therapy. 

Redelman-Sidi and colleagues characterized 45 cancer and HCT patients with 2009H1N1v [32]. They responded well to oseltamivir and had mild respiratory symptoms. However, no patient in this cohort was less than 5 months post transplantation. The most susceptible time for both infection and prolonged shedding appears to be within the first 100 days after transplantation due to immunosuppression. 

 A recent troubling report described the first documented person to person transmission of oseltamivir-resistant 2009H1N1v in an inpatient stem-cell transplant unit [33]. Eleven patients were infected with 2009H1N1v, ten strains of which were genetically related. Eight of the ten displayed the identical H274Y mutation. 

## 4. Treatment Options for Influenza Infection in the Immune Compromised Host

Limited data exists from randomized controlled trials on the utility and correct duration of use of licensed antivirals in immunocompromised individuals. 

## 5. Neuraminidase Inhibitors

At present, the predominant class of antiviral used for the treatment and prophylaxis of influenza are the neuraminidase inhibitors (NAIs) which interfere with the release of IFV particles from infected cells, preventing the spread of infection to other cells. The licensed NAI's include oseltamivir, inhaled zanamivir, and recently under Emergency Use Authorization, IV peramivir. IV zanamivir had been available through an Investigational New Drug Application with the U.S. FDA [34].

The antiviral treatment options for immunocompromised patients are empiric and tailored to the particular strain and its known sensitivity. As discussed above, immunocompromised patients infected with IFV can have prolonged infections lasting for more than five days [35]. Thus longer duration NAI treatment is usually required as cessation of shedding is the desired primary outcome. The specific duration of treatment is often empiric. While NAI's are well tolerated, postlicensing reports have indicated that zanamivir may cause cough, bronchospasm, or even death in patients with preexisting pulmonary disease. Hence, this antiviral is should be used with caution in patients with serious underlying respiratory diseases [36].

Oseltamivir is recommended for infection caused by 2009H1N1v in the immunocompetent host, as this virus is typically resistant to the adamantanes [37], in contrast to the previous 2008-2009 seasonal H1N1 strain, which was characterized by oseltamivir resistance but susceptible to amantadine [38]. As the development of resistance is more complex in immunosuppressed individuals, the U.S. Centers for Disease Control (CDC) has recommended that patients with suspected or confirmed oseltamavir resistant 2009H1N1v be treated with zanamivir [39]. If inhaled zanamivir is contraindicated or not well tolerated, then IV zanamivir is be available for compassionate use from its manufacturer via an emergency IND application to the FDA. The CDC also recommends that patients infected with IFV suspected or documented to have H274Y mutation should not be treated with peramivir. As clinical isolates expressing the oseltamivir resistance-associated substitution H274Y demonstrate reduced peramivir susceptibility in vitro. Of note, a recent report examining IFV isolates from 2008–2010 found 28 resistant to both the adamantanes and oseltamivir [40]. The emergence of dual resistant virus is obviously of great public health concern. 

 The optimal therapy for severely immunosuppressed patients with oseltamivir-resistant 2009H1N1v has not been clearly defined. Some severely immunosuppressed patients with 2009H1N1v have been treated with a combination of IV zanamivir and aerosolized ribavirin [41] or IV zanamivir monotherapy No controlled studies however have confirmed the efficacy of this combination approach [42]. A study of 541 patients with confirmed IFV investigated the use of combination therapy (zanamivir and oseltamivir) versus either oseltamivir or zanamivir monotherapy [43]. For the primary endpoints of declining viral load and time to alleviation of symptoms, combination therapy was less effective than oseltamivir monotherapy and not significantly more effective than zanamivir monotherapy. 

## 6. Clinical Investigational Treatments for Influenza Other Than NAI's

Favipiravir (T705, Toyama Chemical) is an investigational antiviral drug that functions as a nucleotide analog and inhibitor of viral RNA polymerase (PB1, PB2, and PA) [44, 45]. Preclinical studies by Itoh and colleagues suggest that favipiravir is active against pandemic H1N1 strains both in vitro and in vivo [46]. 

A recent study demonstrated that favipiravir was effective against oseltamivir-resistant seasonal and pandemic-viruses in vitro [47]. In addition, the drug has potent activity against H5N1 IFV in vivo [48]. Favipiravir, alone or in combination with licensed NAIs, is being investigated in Phase II clinical studies for the treatment of influenza. There are currently no reports of the use of favipiravir in immunocompromised patients.

DAS181 (Fludase, NexBio Inc.) is a recombinant fusion protein with sialidase activity and carrying a cationic sequence tag on the C-terminus [49]. This drug selectively cleaves sialic acids from host cells, rendering them inaccessible to IFV, which seeks sialic acid as its receptor.

DAS181 has activity against numerous seasonal IFV strains in vitro and in vivo as well as highly pathogenic avian influenza strains (H5N1) [50] and against the 2009H1N1v pandemic strains in vitro, in vivo, and ex vivo [51]. It also has antiviral activity against clinical IFV isolates with the H274Y mutation [52]. DAS181 is currently in phase 2 trials for the treatment of influenza in immunocompetent subjects. 

## 7. Parainfluenza

Human PIVs account for a high proportion of pediatric respiratory infections, including upper respiratory tract infection (URTI), laryngotracheobronchitis (croup), bronchiolitis, and pneumonia [53]. Human PIVs are divided into 4 types, with infections from types 1 and 3 accounting for most disease. Human PIV is the major cause of croup (type 1 is most frequent, followed by type 3 and type 2). Of the PIV's PIV3 is the most common pathogen. Acute respiratory infections cause up to 18% of all admissions to pediatric hospitals, and PIV can be detected in 9 to 30% [54]. There are more than 5 million lower respiratory tract infections in children younger than 5 years each year in the United States, and PIV is found in as many as one-third of them [55, 56]. Each year in the United States, between 500,000 to 800,000 cases of respiratory infection in persons younger than 18 years result in hospitalizations, of which approximately 12% display PIV infection [57]. Although in the immunocompetent host, mortality from PIV is rare, the mortality rate in immunocompromised patients is much higher. 

 PIV is among the respiratory viruses most common in the transplant population. Clinical presentation in the immunocompromised population often differs from that of other respiratory viruses, such as respiratory synctial virus (RSV). In one study, patients with PIV-3 presented with upper respiratory tract infections (URI's) or were asymptomatic [58], contrasting with other respiratory infections which typically present symptomatically. Recent studies document that PIV infections can occur in up to 18% of HCT patients during the first 100 days, and progress from URI to pneumonia in 18 to 44% of patients [59]. These reports suggest that PIV is more common than RSV or IFV and is a significant cause of mortality and morbidity in the transplant population. Death from PIV in HCT can occur in 25 to 45% of infected patients within 30 days after the diagnosis of lower respiratory tract disease. In contrast to IFV, PIV infections occur all year in HCT [60, 61]. PIV infection also occurs following SOT. It has been suggested that PIV infection causes significant morbidity and may be a major factor contributing to the poor prognosis of lung transplant recipients [62]. In a large study, 5.3% of lung transplant recipients were diagnosed with PIV infection using bronchoalveolar lavage or transbronchial biopsy [63]. Lower respiratory tract involvement was reported to occur in 10 to 66% of infected patients. Although the time to development of complications from respiratory viral infections post lung transplantation is variable, most PIV infections are described in the first year. Up to 10% of lung transplant patients develop acute respiratory failure requiring mechanical ventilation following documented PIV infection [64]. Death following the development of PIV induced pneumonia occurred in approximately 35% of patients receiving allografts following myeloablative conditioning [63]. Outbreaks of parainfluenza infection in transplant centers can result in significant mortality due transmission to other patients. A recent described outbreak of PIV-3 in 13 HCT patients resulted in the death of 5 (38.5%), with all having lower tract disease and 4 unsuccessfully treated with ribavarin [65]. 

## 8. Treatment Options for PIV Infection in the Immunocompromised Host

Treatment or prevention options for patients with PIV are limited as there are no approved antivirals or vaccines. Ribavirin has shown both in vitro and in vivo activity against PIV [66]. There have been numerous case reports of the use of this drug against PIV in transplant patients. In one study, only two of five PIV infections after HCT improved with oral ribavirin [67, 68]. Reduction in mortality with aerosolized Ribavirin, with or without immunoglobulin therapy (IVIG), was observed in HCT transplant patients with PIV-3 induced pneumonia [69]. In a cohort of 7 subjects with PIV post lung or heart-lung transplant, a combination approach was used utilizing ribavarin, corticosteroids, and intravenous immunoglobulin G [69]. This study suggested that the use of triple therapy resulted in slower declines in lung function (FEV_1_), compared to historical controls. 

## 9. Clinical Investigational Treatments for PIV

As PIV also uses sialic acids as receptors [70], the host directed approach of DAS181, is being investigated for activity against this pathogen. DAS181 effectively inhibits PIV in multiple cell lines, models of the human airway epithelium, and in vivo animal models [71]. 

A recent report described a 64-year-old female post HCT for acute myeloid leukemia (AML) developed progressive PIV-3 infection documented by direct fluorescent antibody test (DFA) and accompanied by worsening pulmonary status requiring supplemental oxygen [72]. The patient demonstrated PIV-3 shedding for approximately 6 weeks and was treated with DAS181 for three days. Three days after the last dose of treatment, respiratory symptoms and pulmonary function improved and the patient's nasal swab became negative by DFA. PCR of the nasal swabs revealed an over two log drop in virus levels. In-vitro inhibition of the patient's virus by DAS181 was also demonstrated. This patient's lung status improved without requiring supplemental oxygen. However, this patient passed away 12 days later due to relapse of AML. 

A live attenuated PIV-3 vaccine has been tested in children and adults and was found to be safe but was found to be nonimmunogenic in individuals who were seropositive prior to immunization [73, 74]. However, this approach has not yet been tested in immunocompromised patients where vaccine induced antibody responses may be suboptimal compared to immuncompetent individuals. 

## 10. Conclusions

An area of great unmet medical need is the treatment of respiratory viral infections in immunocompromised hosts. During the first month post transplantation, vaccinations appear to be limited in their ability to prevent IFV and no vaccine exists for PIV. For IFV, although effective antivirals exist to treat infections in the immunocompetent host, their utility in this high-risk population is poorly defined and dependent on the susceptibility of the virus. The rate of drug resistance selection appears to be higher in immunocompromised patients than in the immune competent population and the transmission of multidrug resistant virus remains a major public health concern. In the case of PIV, no effective treatment modalities are currently available. Immunocompromised patients exemplify the most severe complications associated with these respiratory infections. New effective therapies for IFV and PIV in these high-risk patients remains an important public health priority. 

## Figures and Tables

**Table 1:** Similarities and differences between IFV and PIV infections in the immunocompromised host.

	Significant morbidity and mortality	RNA virus	Respiratory receptor	Peak incidence	Vaccination during posttransplant period	Antivirals	Antiviral resistance
Influenza	Yes	Yes	Sialic acids	Seasonal	Yes (reduced efficacy)	Yes (unproven efficacy)	Yes
Parainfluenza	Yes	Yes	Sialic acids	Perennial	None licensed	No	N/A
